# Distinct Hole and Electron Transport Anisotropy in Ambipolar Nickel Dithiolene‐Based Semiconductor

**DOI:** 10.1002/anie.202512609

**Published:** 2025-09-03

**Authors:** Masatoshi Ito, Tomoko Fujino, Toshiki Higashino, Mafumi Hishida, Hatsumi Mori

**Affiliations:** ^1^ The Institute for Solid State Physics The University of Tokyo 5‐1‐5 Kashiwanoha Kashiwa Chiba 277‐8581 Japan; ^2^ Department of Chemistry and Life Science Yokohama National University Tokiwadai Hodogaya 79‐5 Yokohama Kanagawa 240‐8501 Japan; ^3^ National Institute of Advanced Industrial Science and Technology 1‐1‐1 Higashi Tsukuba Ibaraki 305‐8565 Japan; ^4^ Department of Chemistry Faculty of Science Tokyo University of Science 1–3 Kagurazaka Shinjuku Tokyo 162‐8601 Japan

**Keywords:** Ambipolar semiconductor, Anisotropy, Crystalline thin film, Field‐Effect transistor, Intermolecular interaction

## Abstract

Understanding anisotropic charge transport in molecular semiconductors is crucial for device optimization, yet its intricate dependence on orbital‐specific intermolecular interactions and molecular packing remains a challenge, especially in ambipolar systems. In ambipolar semiconductors, where both holes and electrons participate in conduction, distinct molecular orbitals prompt a critical inquiry: can orbital variations result in coexisting yet distinct anisotropic transport properties within a single component? We confirm this possibility by demonstrating that the air‐stable nickel dithiolene, **Ni(4OPr)**, exhibits such behavior. Despite its herringbone stacking implying a two‐dimensional electronic structure, **Ni(4OPr)** uniquely exhibits distinct intermolecular interactions for hole (HOMO‐to‐HOMO; HOMO = highest occupied molecular orbital) and electron (LUMO‐to‐LUMO; LUMO = lowest unoccupied molecular orbital) transport. Crucially, this leads to highly anisotropic hole transport pathways, while electron pathways are remarkably isotropic, demonstrating a stark contrast in their transport anisotropies. Leveraging the high crystallinity, grazing‐incidence wide‐angle X‐ray scattering (GIWAXS) determined in‐plane molecular orientation. This enabled experimental verification of distinct anisotropic hole and electron transport, directly governed by orbital‐specific intermolecular interactions, in an ambipolar molecular semiconductor. Our findings, demonstrating coexisting yet distinct anisotropic transport properties for both carriers within a single component, significantly advance the understanding of ambipolar molecular semiconductors and broaden their scope for future device applications.

## Graphical abstract

Simultaneous yet distinct anisotropic transport of holes and electrons is demonstrated within a single‐component ambipolar semiconductor. Utilizing an air‐stable ambipolar semiconductor, we elucidated frontier orbital‐specific intermolecular interactions governing the factor for this transport anisotropy, guiding the development of next‐generation anisotropic electronic devices.

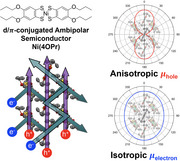

## Introduction

Molecular semiconductors, including π‐conjugated small molecules,^[^ ^1^, ^2^, ^3^, ^4^, ^5^, ^6^ ^]^ are distinguished from inorganic semiconductors (e.g., silicon) by attributes such as mechanical flexibility and solution processability. These benefits, coupled with the ease of obtaining structural insights distinct from polymer materials, have driven extensive academic and industrial research into molecular semiconductors in recent decades.^[^ ^7^ ^]^ Unlike inorganic semiconductors that form rigid crystals via strong covalent bonds, molecular semiconductors form more flexible crystals, where geometrically anisotropic planar molecules pack densely through anisotropic intermolecular interactions such as van der Waals forces and π–π stacking. These interactions directly dictate the dimensionality of the electronic structure. This is because intermolecular frontier orbital interactions, responsible for carrier conduction, depend on the crystallographic direction, thereby reflecting molecular packing. Consequently, the carrier transport properties of small‐molecule semiconductors exhibit anisotropy, corresponding to electronically relevant packing arrangements. This anisotropy is less pronounced in polymeric semiconductors, where polydispersity typically leads to disordered molecular assemblies with reduced directional variations in intermolecular interactions. Therefore, the intrinsic relationship between molecular packing structure and carrier transport characteristics is distinctive to small‐molecule semiconductors.^[^ ^8^, ^9^ ^]^

In principle, carrier transport anisotropy can manifest not only in unipolar semiconductors,^[^ ^10^, ^11^, ^12^, ^13^, ^14^, ^15^, ^16^ ^]^ which conduct either holes or electrons exclusively, but also in ambipolar semiconductors,^[^ ^17^ ^]^ which can conduct both carriers within a single component. An intriguing and ideal possibility for ambipolar small‐molecule semiconductors is the coexistence of distinct anisotropic transport properties for holes and electrons within a single‐component, single‐crystal structure. This phenomenon arises because holes and electrons are transported via different frontier orbitals (HOMO and LUMO, respectively). The shapes and symmetries of these orbitals then lead to distinct modes of carrier mobility governed by anisotropic intermolecular interactions. Elucidating the structural details underlying this anisotropy in both hole and electron transport in ambipolar small‐molecule semiconductors would deepen our understanding of conduction mechanisms. This advancement will potentially enhance device performance by optimizing channel orientation and improving carrier separation or recombination processes. This may also pave the way for optical devices featuring luminescence patterns that differ from those of conventional light‐emitting devices, representing a promising property for ambipolar semiconductor materials. However, despite the development of hundreds of ambipolar small‐molecule semiconductors,^[^ ^17^ ^]^ experimental investigations of anisotropic transport in these materials remain limited compared to unipolar small‐molecule semiconductors such as rubrene^[^ ^10^ ^]^ and pentacene.^[^ ^11^, ^12^, ^13^, ^14^, ^15^, ^16^ ^]^ While theoretical studies have modeled the anisotropic mobilities of holes and electrons,^[^ ^18^, ^19^, ^20^ ^]^ experimental validation of such transport in ambipolar molecular semiconductors has, to our knowledge, not yet been achieved. This lack of experimental progress is primarily hindered by two key challenges. First, there is the difficulty of obtaining large‐area, highly crystalline thin films suitable for in‐plane angular‐dependent field‐effect transistor (FET) measurements with multiple channel directions within a single domain. Second, the electronic structure presents an issue: materials with strictly one‐dimensional (1D) molecular packing arrangements exhibit a 1D electronic structure. Such 1D systems are sensitive to molecular defects, which often destabilize the FET operation. Realizing more robust, higher‐dimensional electronic structures remains a significant hurdle.

**Figure 1:**
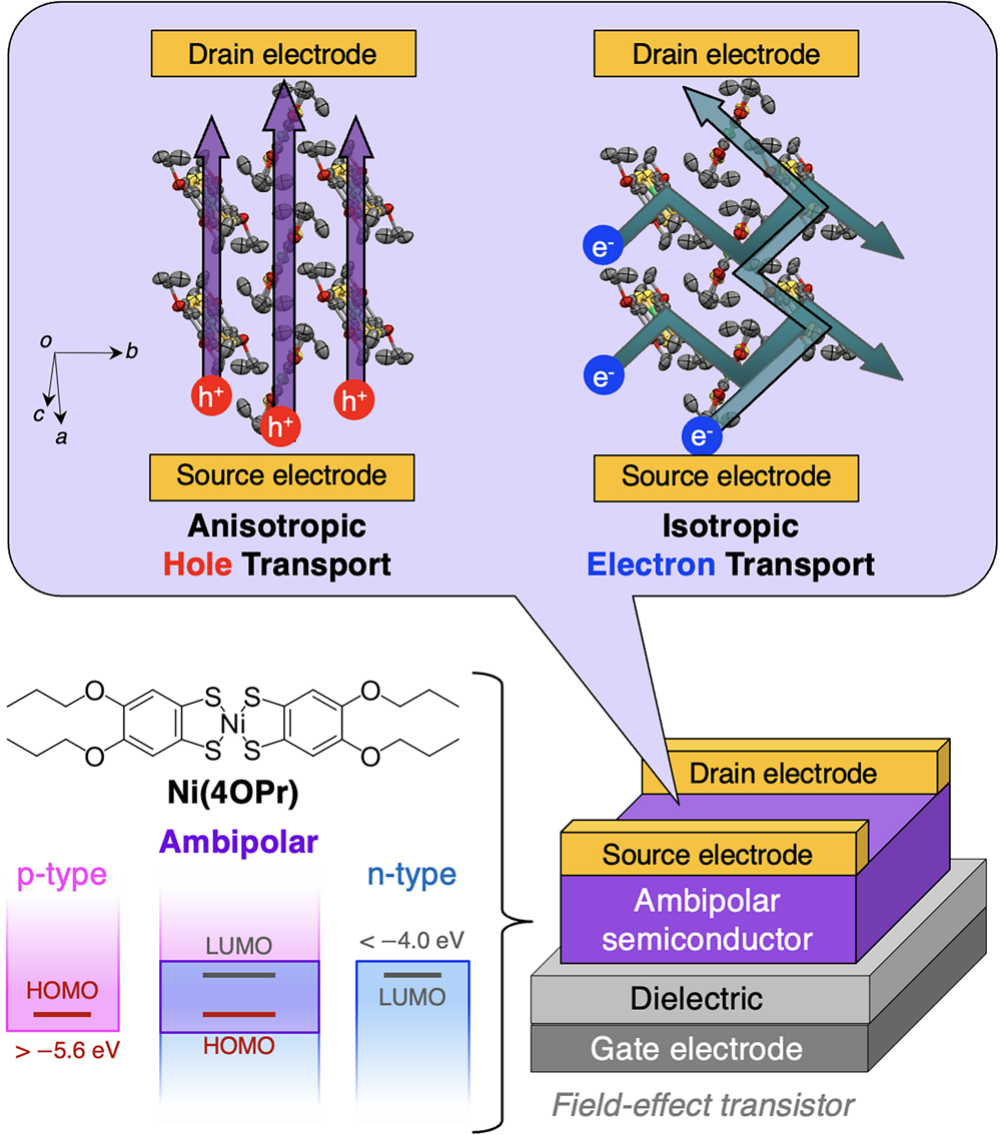
(Top) Demonstration of anisotropic hole conduction and isotropic electron conduction in ambipolar molecular semiconductor **Ni(4OPr)**. (Bottom) Structural formula and electronic structure of **Ni(4OPr)**, with HOMO and LUMO energy levels of −5.3 and −4.3 eV, respectively, indicating stable expression of ambipolar field‐effect characteristics in air.

To address these challenges, we utilized our recently developed neutral nickel bis(dithiolene) complex, **Ni(4OPr)**, a solution‐processable ambipolar molecular semiconductor stable under atmospheric conditions (Figure 1).^[^ ^21^ ^]^ Its deep LUMO level and narrow HOMO–LUMO gap, arising from molecular orbitals that combine d electrons from the central metal with π electrons from the ligand, facilitate stable ambipolar carrier transport in air—a property not readily achievable with conventional small π‐conjugated molecules. The planar X‐shaped molecular geometry, imparted by its four *n*‐propoxy substituents, promotes close intermolecular contacts where the substituents are positioned near the d/π‐planes of neighboring molecules. This leads to a herringbone molecular stacking structure, establishing a two‐dimensional (2D) electronic structure with conduction pathways in both columnar and transverse directions. Additionally, **Ni(4OPr)** forms highly crystalline thin films spanning several hundred micrometers via blade coating, making it ideal for investigating anisotropic carrier transport under atmospheric conditions.^[^ ^22^ ^]^

In this study, we report the first experimental observation of distinct anisotropic transport for both holes and electrons within a single‐component ambipolar semiconductor. Using **Ni(4OPr)** thin films with crystal orientation characterized by grazing‐incidence wide‐angle X‐ray scattering (GIWAXS), we successfully demonstrated distinct anisotropies in hole and electron transport within a small‐molecule‐based ambipolar semiconductor. This unprecedented finding provides fundamental insight into conduction mechanisms, supports the establishment of molecular design guidelines, and broadens the applicability of ambipolar molecular semiconductors.

## Results and Discussion

### Theoretical Simulation for Anisotropic Carrier Transport Properties

**Figure 2:**
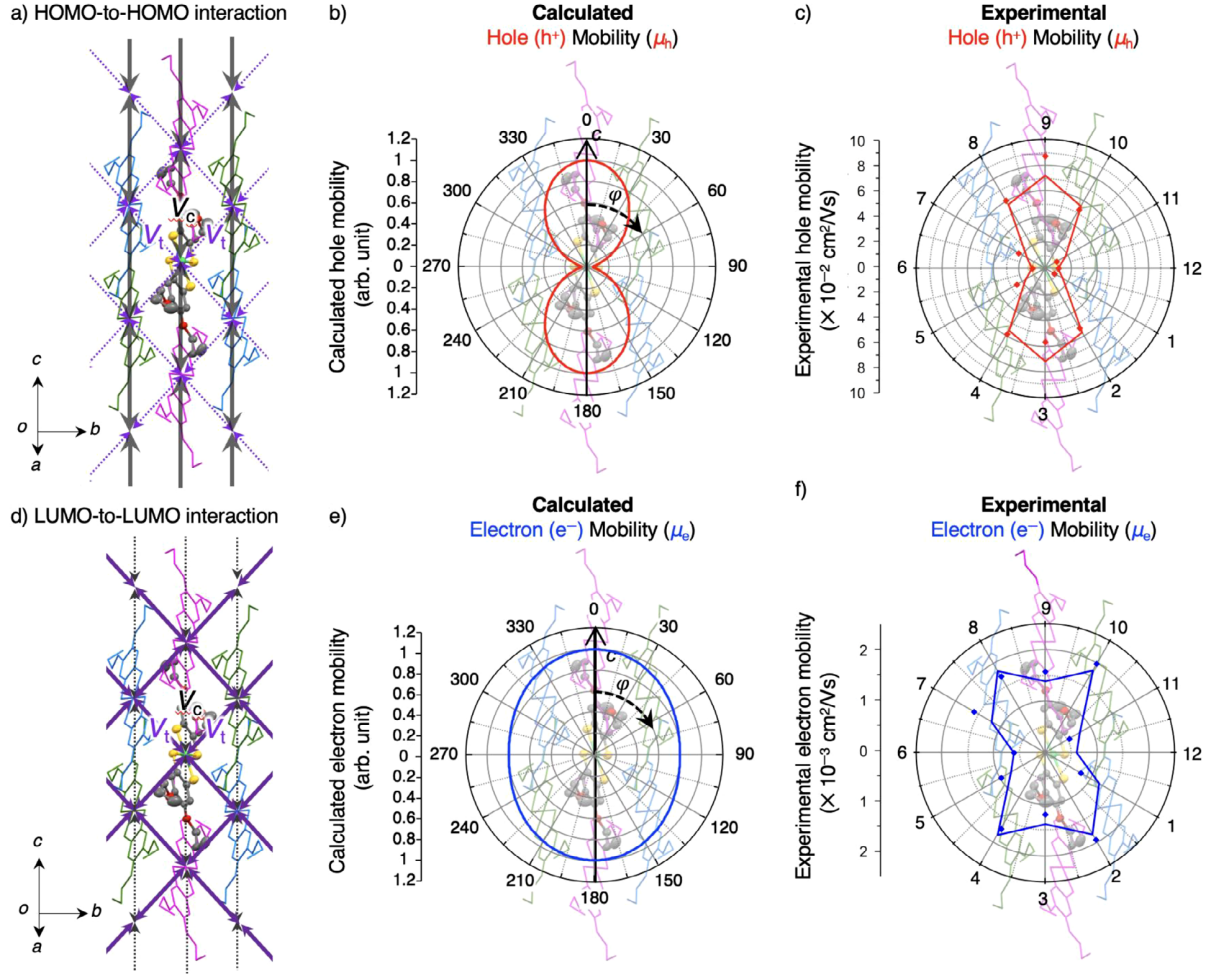
a) Dominant 1D HOMO‐to‐HOMO interaction network responsible for hole conduction in the herringbone stacking form of **Ni(4OPr)**. Calculated HOMO‐to‐HOMO electron couplings between neighboring molecules—colored pink, green, and aqua for the columnar and two transverse orientations, respectively—are 11 meV along the column direction (*V*_c_; black arrow) and 6.6 meV for the transverse direction (*V*_t_; purple arrow). b) Simulated in‐plane (*bc*‐plane) angular (*φ*) dependence of hole mobility, where the *c*‐axis is defined to be *φ* = 0°. Ratio of maximum to minimum hole mobility: 20. Maximum mobility is standardized as 1. c) Experimental in‐plane angular dependence of hole mobility. Points in the plot indicate the actual measured mobility for each channel, while the lines connect the average values of two channels oriented in the same direction (e.g., 12–6, 1–7, and 2–8). The highest hole mobility of 8.7 × 10^−2^ cm^2^ V^−1^ s^−1^ with an on/off ratio of ∼10^5^ was observed at Channel 9. Ratio of maximum to minimum hole mobility: 11. d) Dominant 2D LUMO‐to‐LUMO interaction network responsible for electron conduction. Calculated LUMO‐to‐LUMO electron couplings between neighboring molecules along the column direction (*V*_c_; black arrow) and transverse direction (*V*_t_; purple arrow) are 6.6 and 17 meV, respectively. e) Simulated in‐plane (*bc*‐plane) angular dependence of electron mobility. Ratio of maximum to minimum hole mobility: 1.2. Maximum mobility is standardized as 1. f) Experimental in‐plane angular dependence of electron mobility. Points in the plot indicate the actual measured mobility for each channel, while the lines connect the average values of two channels oriented in the same direction (e.g., 12–6, 1–7, and 2–8). The highest electron mobility of 2.0 × 10^−3^ cm^2^ V^−1^ s^−1^ with an on/off ratio of ∼10^4^ was observed at Channel 10. Ratio of maximum to minimum hole mobility: 3.7.

Prior to experimental device fabrication, we theoretically examined anisotropic carrier transport in herringbone‐packed **Ni(4OPr)** crystals. We calculated the in‐plane angular dependence of hole and electron mobilities within the *bc*‐plane, utilizing first‐principles quantum mechanical calculations combined with the Marcus–Hush theory (Figure S1).^[^ ^23^ ^]^ To estimate angle‐dependent carrier transport characteristics, we first employed the Amsterdam Density Functional program^[^ ^24^ ^]^ at the GGA:PW91/TZP level^[^ ^25^, ^26^ ^]^ to calculate electron coupling between neighboring HOMOs required for simulating carrier mobility. We determined electron couplings of 11 meV along the stacking column (*V*_c_; columnar direction; *c*‐axis; defined as *φ* = 0° in the *bc*‐plan in Figure 2a,b) and 6.6 meV between adjacent columns (*V*_t_; transverse direction) (Figure 2a). Based on these values, the angular dependence of hole mobility in the *bc*‐plane was simulated using the Marcus–Hush theory (see Supporting Information for the details), revealing a peak along the *c*‐axis and a trough along the *b*‐axis (Figure 2b). The maximum‐to‐minimum hole mobility ratio reached 20, indicating strong anisotropy. Notably, this anisotropy exceeds the ∼1.7 ratio of the HOMO‐to‐HOMO couplings between columnar and transverse directions, suggesting that the dominant HOMO‐to‐HOMO interactions along the columns effectively suppress hole transport between columns (Figure 2a). From a materials design perspective, this highlights the importance of aligning the channel with the maximum conduction axis (i.e., columnar direction) to maximize hole mobility.

We similarly examined electron transport, determining LUMO‐to‐LUMO couplings of 6.6 meV along the columnar direction and 17 meV for the transverse direction (Figure 2d). Although the stronger intercolumn coupling might suggest greater anisotropy, simulated electron mobility peaked along the *c*‐axis and was minimized along the *b*‐axis (Figure 2e). The ratio of maximum‐to‐minimum electron mobility was only 1.2, indicating comparatively isotropic transport. Despite the LUMO‐to‐LUMO coupling for the transverse direction being ∼2.5 times larger than the columnar coupling, the overall electron transport was more 2D and less directionally skewed. This challenges the prevailing assumption that frontier orbital coupling anisotropy directly corresponds to mobility anisotropy.^[^ ^9^ ^]^ In **Ni(4OPr)**, robust transverse LUMO‐to‐LUMO interactions facilitate a 2D electron conduction pathway (Figure 2d) that competes with the columnar conduction pathway. Consequently, enhancing transverse interactions within the herringbone packing arrangement can reduce carrier transport anisotropy, and conversely, weakening them can increase it, a principle widely relevant to molecular semiconductors. Overall, these calculations indicate that in **Ni(4OPr)**, HOMO‐to‐HOMO interactions predominantly support anisotropic conduction along the columns, while LUMO‐to‐LUMO interactions facilitate more uniform 2D electron conduction, leading to simultaneous distinct anisotropies for holes and electrons.

### Device Fabrication and In‐Plane Molecular Orientation Analysis

**Figure 3:**
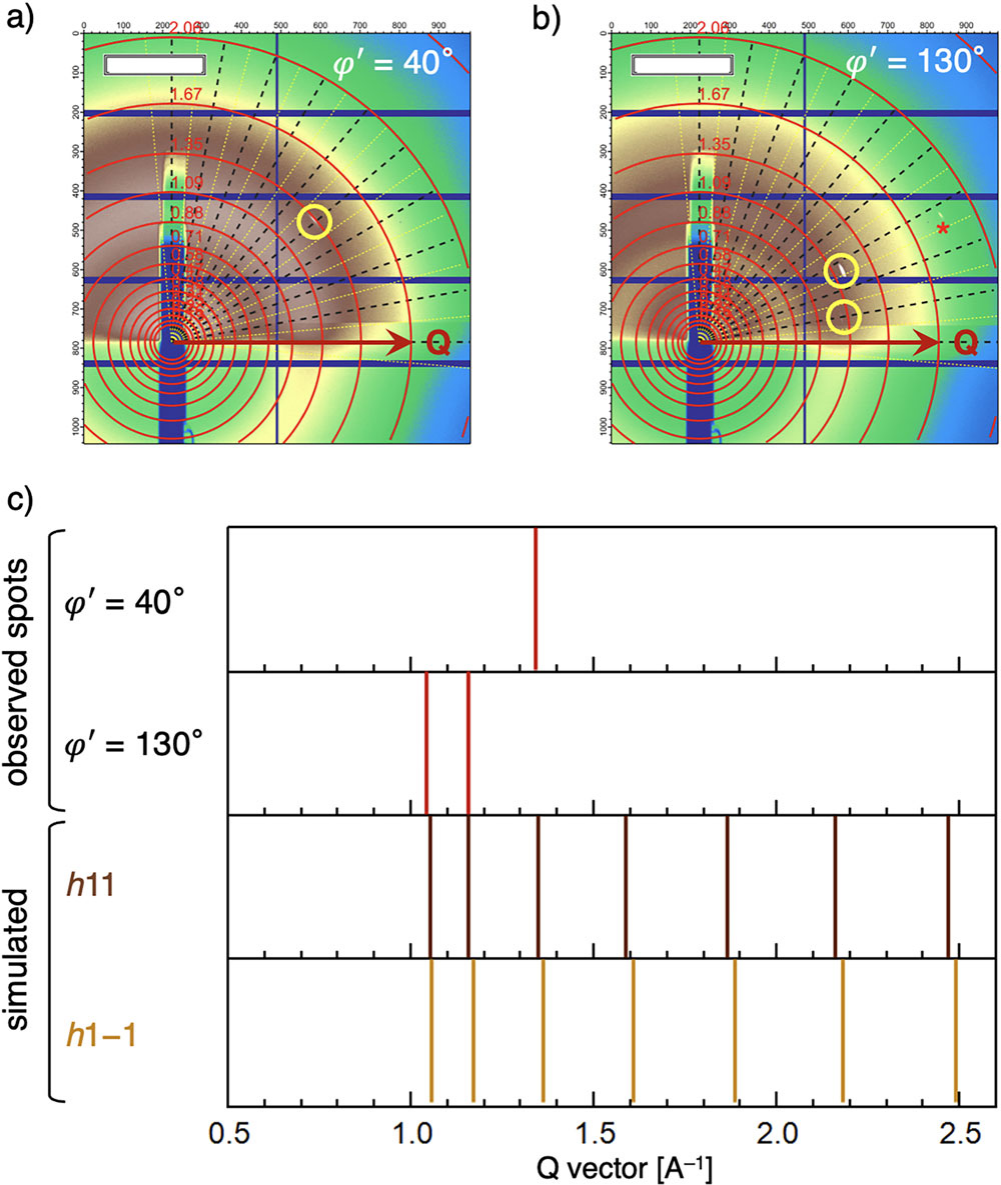
GIWAXS profiles at 40° a) and 130° b) X‐ray incidence angles. Yellow circles highlight diffraction spots, suggesting high crystallinity of the thin films. The asterisk in (b) indicates a diffraction spot derived from the SiO_2_ substrate. c) Attribution of the *h*11 and *h*1−1 diffraction spots observed at incidence angles *φ*' of 40° and 130°, respectively (or vice versa), based on their scattering vector (Q) values. See details in Figures S4–S10 and Table S1.

To explore anisotropic carrier transport, we fabricated FETs using highly crystalline **Ni(4OPr)** thin films and determined their in‐plane molecular orientations. Given the tendency of **Ni(4OPr)** to form extensive single‐crystal domains in solution‐processed thin films,^[^ ^21^ ^]^ we optimized the blade‐coating^[^ ^22^ ^]^ parameters by increasing solution concentration and sweep speed (see Supporting Information for the details) to produce crystalline films suitable for assessing the angular dependence of carrier mobility within a single domain (typically ∼500 × ∼500 µm^2^, see Figures S5 and S10a). High crystallinity and a smooth surface were further confirmed by GIWAXS (exhibiting prominent diffraction peaks; see Figures 3a,b and S6) and atomic force microscopy (AFM) measurements (Figure S3), respectively. To precisely determine molecular orientation, we exclusively used single‐domain substrates and eliminated surrounding areas. Bottom‐gate, top‐contact FETs with Au electrodes arranged in a fan‐shaped pattern at 30° intervals were fabricated (Figures S5 and S10). We defined the in‐plane direction to the blade‐coating direction as *φ*' = 0° (Figure S4), and in‐plane transport measurements were subsequently performed at 30° increments (i.e., Δ*φ*' = 30°), covering angles from *φ*' = 0° (Channel 12) to *φ*' = 330° (with Channels 1, 2, etc., corresponding to 30°, 60°, and so on). GIWAXS was employed to determine the corresponding in‐plane molecular orientation of the films. GIWAXS profiles were acquired using an auto‐rotation stage (Figure S4a), rotating the sample in 10° increments of *φ*' (i.e., Δ*φ*' = 10°). The resulting sharp diffraction spots confirmed high film crystallinity (Figures 3a,b and S5). These spots were indexed as (*hkl*) based on their scattering vector (Q) values for each incident angle *φ*'. This analysis enabled us to determine the in‐plane molecular orientation angle (*φ*, as defined in Figure S1) relative to the *φ*' = 0 direction and to correlate it with the FET channel direction (*φ*', as defined in Figure S4b). In blade‐coated **Ni(4OPr)** films, the (*h*00) planes preferentially align parallel to the substrate,^[^ ^21^ ^]^ simplifying the analysis of the in‐plane orientation of the orthogonal *b*‐ and *c*‐axes within the monoclinic crystal system. For instance, in a representative sample (Figures 3c and S7; Table S1), strong diffraction spots were observed for (*h*11) at an incidence angle of approximately 40° and for (*h*1−1) at 130°—i.e., perpendicular to 40°—or vice versa. In contrast, spots associated with (*h*0 ± 1) or (*h*0 ± 2) appeared around 10°, 150°, and 170°.^[^ ^23^ ^]^ This analysis revealed that the *b*‐axis (*φ* = 90°) was nearly parallel to the blade‐coating direction *φ*' = 90°, with the *c*‐axis oriented approximately perpendicularly (Figure S5). It is noted that the *b*‐axis likely aligns closely with the blade‐coating direction (i.e., film growth direction), though the angular offset varies, sometimes by tens of degrees. This observed tendency may be related to the interfacial meniscus formed during the blade‐coating process, which could be an intriguing topic for future studies on solution‐based processes.

### Anisotropic In‐Plane FET Characteristics

We present the results of FET characterizations conducted at 30° intervals on single‐domain **Ni(4OPr)** thin films. Owing to the sensitivity of **Ni(4OPr)** electron transport to ambient moisture, initial measurements were performed under vacuum to minimize external influences. For hole transport, channels oriented along the *c*‐axis (Channels No. 3 and 9 in Figure 2c) exhibited higher mobilities, whereas those aligned closer to the *b*‐axis (Channels No. 6 and 12) showed lower mobilities. The on/off ratios for all channels were on the order of 10^4^–10^5^, consistently among the highest levels for ambipolar molecular semiconductors. Specifically, Channel 9 demonstrated the highest hole mobility of 8.7 × 10^−2^ cm^2^ V^−1^ s^−1^, with a maximum‐to‐minimum mobility ratio of 11 (Figure 2c and Table 1). These findings are consistent with theoretical predictions and confirm that strong HOMO‐to‐HOMO interactions along the columnar direction led to pronounced anisotropic hole transport in functional FET devices.

**Table 1:** Hole/electron mobilities (*μ*), threshold voltages (*V*_th_), and on/off ratios for each channel of the FET shown in Figure 2.

	Hole transport			Electron transport		
No.	*μ* (cm^2^ V^−1^ s^−1^)	*V*_th_ (V)	On/off	*μ* (cm^2^ V^−1^ s^−1^)	*V*_th_ (V)	On/off
1	8.3×10^−3^	−36	10^5^	8.1×10^−4^	80	10^4^
2	5.4×10^−2^	−13	10^5^	2.0×10^−3^	95	10^5^
3	5.7×10^−2^	−9.1	10^5^	1.2×10^−3^	89	10^3^
4	5.9×10^−2^	−5.6	10^5^	1.7×10^−3^	101	10^5^
5	2.6×10^−2^	−2.1	10^5^	9.9×10^−4^	102	10^4^
6	9.7×10^−3^	−6.6	10^4^	6.1×10^−4^	110	10^3^
7	2.4×10^−2^	2.5	10^5^	1.6×10^−3^	107	10^4^
8	6.1×10^−2^	6.3	10^5^	1.7×10^−3^	107	10^4^
9	8.7×10^−2^	−4.1	10^5^	1.6×10^−3^	101	10^5^
10	5.3×10^−2^	1.3	10^5^	2.0×10^−3^	96	10^4^
11	1.0×10^−2^	−19	10^4^	5.5×10^−4^	88	10^4^
12	1.0×10^−2^	−23	10^4^	(6.1×10^−4^)	(110)	(10^3^)

In examining electron transport, channels near the *c*‐axis, where hole mobilities were relatively high, also displayed higher electron mobilities. The highest electron mobility of 2.0 × 10^−3^ cm^2^ V^−1^ s^−1^ was observed in Channel 10 under vacuum, with a maximum‐to‐minimum mobility ratio of 3.7 (Figure 2f and Table 1). Thus, electron mobility exhibited a weaker directional dependence compared to hole mobility, showing a slightly dominant preference for conduction along the transverse direction over adjacent column directions. The on/off ratios were on the order of 10^3^–10^5^, which is also consistently high for ambipolar molecular semiconductors.

These trends are consistent with our theoretical simulations, suggesting the occurrence of 2D electron transport through adjacent columns—originating from robust electron coupling between LUMOs—in FET devices.

Similar measurements conducted under ambient conditions replicated the qualitative trends, revealing highly anisotropic hole mobilities alongside relatively isotropic electron mobilities (refer to Supporting Information). A hole mobility of 2.1 × 10^−1^ cm^2^ V^−1^ s^−1^ was achieved in air (Figures S8–S10 and Table S2), significantly surpassing the previously reported maximum hole mobility of 2.0 × 10^−3^ cm^2^ V^−1^ s^−1^ for **Ni(4OPr)** by two orders of magnitude.^[^ ^21^ ^]^ This improvement is primarily due to aligning the FET channel with the optimal conduction direction and fine‐tuning the film growth conditions. Notably, the ratio of maximum‐to‐minimum hole mobility under ambient conditions exceeded 14, highlighting the critical importance of channel orientation in maximizing transport performance in molecular semiconductors. In contrast, electron transport is likely inferior under ambient conditions compared to vacuum, characterized by lower overall mobility and slightly higher threshold voltages. The anisotropy ratio for electrons (approximately 2.5) remained considerably lower than that for holes.

**Figure 4:**
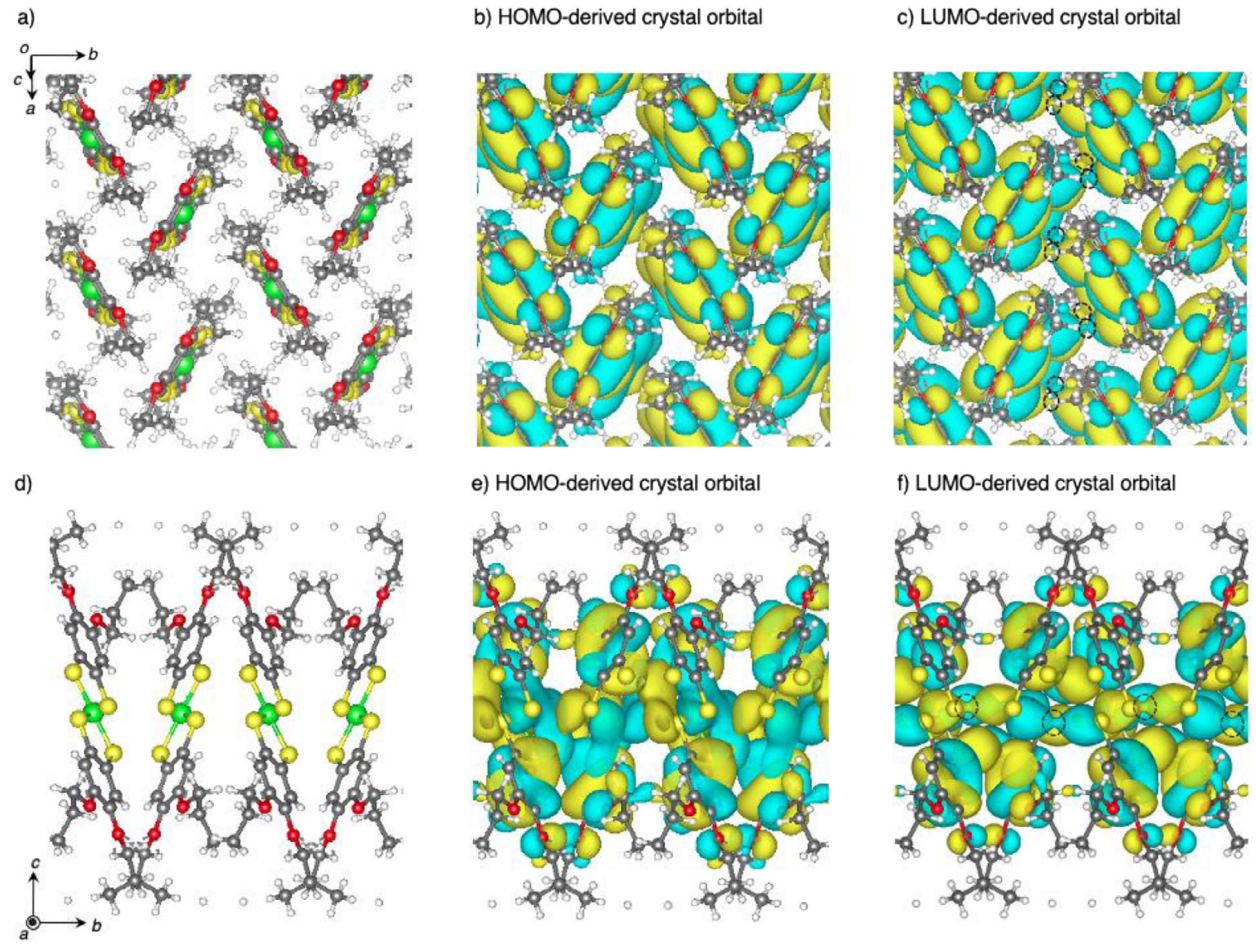
The packing structure of **Ni(4OPr)**, as determined by single‐crystal X‐ray diffraction (XRD), is depicted: a) a view perpendicular to the herringbone 2D plane, and b) a view along the *a*‐axis. DFT‐based crystal orbitals are also presented: the highest occupied crystal orbital (HOCO), derived from the HOMO, at the Γ point (b, e); and the LUCO, derived from the LUMO, at the Γ point (c, f). The in‐phase crystal orbitals overlap between molecules in the transverse direction, as observed in the LUCO (c, f, and Figure S2), as shown with a dashed circle. Atoms were colored as follows: green: nickel atom; yellow: sulfur atom; red: oxygen atom; gray: carbon atom; white: hydrogen atom.

These results demonstrate that **Ni(4OPr)** exhibits coexisting yet distinctly anisotropic transport characteristics for both holes and electrons within a single component and crystal structure, consistent with the different configurations of HOMO‐to‐HOMO and LUMO‐to‐LUMO interactions. Although these experimental trends align with theoretical calculations, the electron mobility in FET devices was somewhat more anisotropic than predicted. This difference may arise from the relatively low electron mobility of **Ni(4OPr)** compared to its hole mobility, which renders it more susceptible to local film quality and contact effects, as well as from the inherent limitations in the computational model. Future investigations could involve the calculation of the LUMO‐derived crystal orbital (lowest unoccupied crystal orbital, [LUCO]). In our simulations, the spatial configuration of the hopping sites was established relative to the central Ni atom, proposing a model where carriers directly hop between Ni centers in neighboring molecules. However, the frontier orbitals of **Ni(4OPr)** are delocalized across the entire molecule, predominantly over the S atoms in the ligands. The orbital shape may significantly impact electron coupling between adjacent molecules (Figure 4). Notably, the LUCO on the S atoms displays in‐phase overlap between molecules in the transverse direction (Figures 4c,f and S2), a feature absent for the LUCO in the columnar direction or for the HOMO‐derived crystal orbitals (Figure 4b,e). This strong LUMO‐to‐LUMO coupling between neighboring columns is potentially driven by the in‐phase LUCO overlap. Since the simulation model designates hopping sites based on the central Ni atom, it does not fully capture this effect, potentially overestimating the electron transfer distance associated with single‐hopping events in the adjacent column direction (Figure S2). Consequently, this may lead to an overestimation of electron mobility in the transverse direction in the calculations, which could account for the observed discrepancy in the degree of electron mobility anisotropy.

## Conclusions

Our experiments confirmed the coexistence of distinct anisotropic transport properties for holes and electrons in the crystalline molecular semiconductor **Ni(4OPr)**. The 2D electronic structure of the material, derived from herringbone packing and the preserved high crystallinity in solution‐coated thin films, facilitated anisotropic carrier transport measurements in FETs alongside GIWAXS‐based in‐plane molecular orientation assessments. We detected considerably higher hole mobility along the stacking column (*c*‐axis), demonstrating strong anisotropy, while electron transport remained relatively isotropic. This distinct behavior is attributed to the contrasting dominance of the HOMO‐to‐HOMO interactions within the columns compared to the LUMO‐to‐LUMO interactions between columns, promoting a 2D electron conduction pathway, as supported by the Marcus–Hush‐based theoretical simulations. Furthermore, understanding the anisotropy of hole conduction allowed us to control and increase the hole mobility by two orders of magnitude compared to the previously reported maximum values;^[^ ^21^ ^]^ the observed hole mobility (2.1 × 10^−1^ cm^2^ V^−1^ s^−1^) is among the highest reported values for an ambipolar molecular semiconductor.^[^ ^17^ ^]^

These results offer critical experimental insights into how intermolecular interaction anisotropy influences device‐level carrier mobility and the effects of channel direction optimization, thereby informing both fundamental research and device engineering. Additionally, our findings underscore a general design principle: enhancing intermolecular interactions within stacking columns promotes highly anisotropic carrier mobility, while strengthening intercolumnar interactions suppresses orientation‐dependent transport in herringbone‐packed molecular semiconductors. The observed coexistence of anisotropic transport in **Ni(4OPr)** highlights a unique attribute of small‐molecule materials, supported by unambiguous structure–property relationships, and potentially expands the application range of ambipolar molecular semiconductors. This study provides a valuable dataset correlating frontier orbital interaction anisotropy with in‐plane mobility anisotropy in FET devices, contributing to refined design principles for ambipolar materials. The simultaneous presence of anisotropic hole and electron conduction within a single component facilitates the optimization of channel orientation to enhance mobility, regulation of carrier flow to improve separation and recombination processes, and investigation of novel light emission phenomena. Consequently, we expect that these findings will accelerate advancements in ambipolar molecular semiconductors and expand their range of technological applications.

## Supporting Information

The authors have cited additional references within the Supporting Information.^[^ ^27^, ^28^, ^29^, ^30^, ^31^, ^32^, ^33^, ^34^, ^35^, ^36^, ^37^, ^38^ ^]^

## Conflict of Interests

The authors declare no conflict of interest.

## Supporting information



Supporting Information

## Data Availability

The data that support the findings of this study are available in the supporting material of this article. Other data are available from the corresponding authors upon reasonable request.
