# Supplementary material for: Distinct Hole and Electron Transport Anisotropy in Ambipolar Nickel Dithiolene‐Based Semiconductor
Source: Angew Chem Int Ed Engl. 2025 Sep 3;64(42):e202512609. doi: 10.1002/anie.202512609 (PMC12518687; doi:10.1002/anie.202512609)
Supplement: Supplementary file 1 — Supporting Information [file ANIE-64-e202512609-s001.pdf]

**Supporting Information**

# Distinct Hole and Electron Transport Anisotropy in Ambipolar Nickel Dithiolene-based Semiconductor

Masatoshi Ito,[a] Tomoko Fujino,*[a,b] Toshiki Higashino,[c] Mafumi Hishida,[d] and Hatsumi Mori*[a]

[a] The Institute for Solid State Physics, The University of Tokyo, Kashiwanoha 5-1-5, Kashiwa, Chiba 277-8581.

[b] Department of Chemistry and Life Science, Yokohama National University, Tokiwadai Hodogaya 79-5, Yokohama, Kanagawa, 240-8501

[c] National Institute of Advanced Industrial Science and Technology, Higashi 1-1-1, Tsukuba, Ibaraki 305-8565

[d] Department of Chemistry, Faculty of Science Division I, Tokyo University of Science Kagurazaka 1-3, Shinjuku, Tokyo 162-8601

## Table of Contents

1. Simulation of Anisotropic Carrier Transport Based on Marcus–Hush Theory ............................... S2
2. Calculation of the Crystal Orbitals of **Ni(4OPr)** ............................................................ S5
3. FET Fabrication and Characterization ............................................................................ S6
4. Determination of the In-Plane Molecular Orientation in FET Thin Films .................................... S8
5. Correlation between FET characteristics under vacuum and crystallographic orientation in the channel directions ............................................................................................................ S10
6. Correlation between FET characteristics in air and crystallographic orientation in the channel directions ..................................................................................................................... S13

## 1. Simulation of Anisotropic Carrier Transport Based on Marcus–Hush Theory

We simulated the in-plane angular dependence of carrier mobility in the two-dimensional conduction plane using the method developed by Han et al. in 2009,[23] which combines first-principles quantum mechanical calculations with the Marcus–Hush theory.[27, 28] We consider the two-dimensional conduction plane parallel to the *bc* plane (Figure S1), where effective intermolecular interactions exist, while ignoring carrier transport along the *a*-axis direction. Within this two-dimensional conduction plane, one molecule is defined as the "central molecule," and its six neighboring molecules are designated as hopping sites ($i$ = 1–6) (Figure S1). Additionally, we define the "basis stack direction" as the direction of the molecular stacking columns (the *c*-axis) in the herringbone structure (Figure S1). The distance between the central molecule and each hopping site, along with the angle between the direction from the central molecule to each hopping site and the basis stack direction, are measured with reference to the central Ni atom. The structural information required for the calculations was taken directly from single-crystal X-ray diffraction data without any additional optimization and was likewise defined based on the central Ni atom coordinates. We used the Amsterdam Density Functional (ADF) program[24] to calculate the electron coupling between the HOMOs and the LUMOs of the central molecule and its hopping sites. The Perdew–Wang gradient-corrected exchange correlation functional[25] and a triple-ζ plus polarization (TZP) basis set composed of Slater-type functions[26] were applied. In this study, we did not compute the reorganization energy $\lambda$, focusing on the relative anisotropy of carrier mobility (we normalized the maximum mobility to 1). This computational approach is both straightforward and widely used in molecular-semiconductor development because it generally reproduces experimental trends well and helps clarify the structure–property relationship. Hereafter, the distance between the central molecule and each hopping site is denoted as $r_i$, the angle between the direction of each hopping site and the ground-stack direction as viewed from the central molecule as $\theta_i$ (Figure S1), and the HOMO-HOMO and LUMO-LUMO electronic couplings of the central molecule and each hopping site $i$ as $V_i$.

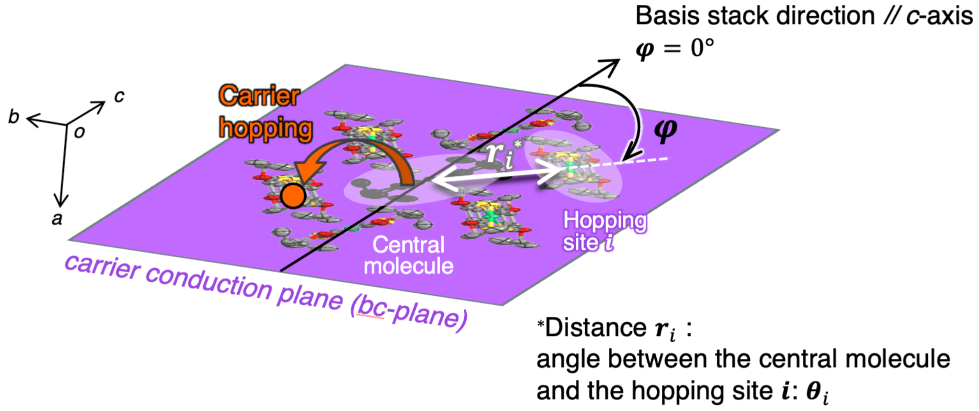


**Figure S1**. Schematic diagram illustrating the assumed two-dimensional carrier conduction plane, the basis stack direction, and definitions of the distances ($r_i$) and angles ($\theta_i$) between the central molecule and the hopping sites.

Under the assumption of carrier transport via a hopping mechanism in a perfect crystal, the electron hopping rate from the central molecule to a hopping site follows the Marcus–Hush equation:

$$W_i = \frac{{V_i}^2}{\hbar}\left(\frac{\pi}{\lambda k_B T}\right)^{1/2} exp(-\lambda/4k_B T) \qquad (1)$$

where $\hbar$ is the Dirac constant, $\lambda$ is the reorganization energy (discussed below), $k_B$ is the Boltzmann constant, and $T$ is the temperature. The hopping probability $P_i$ to each site is then given by:

$$P_i = W_i \Big/ \sum_i W_i \qquad (2)$$

Under the assumption of an uncorrelated random walk and uniform charge motion,[29, 30, 31] the carrier mobility $\mu_\Phi$ in the direction at an angle $\Phi$ from the basis stack axis can be derived from the Einstein relation:

$$\mu_\Phi = \frac{e}{2k_B T}\sum_i W_i {r_i}^2 P_i cos^2(\theta_i - \Phi) \qquad (3)$$

where $e$ is the elementary charge.

The reorganization energy $\lambda$[7, 32] for hole transport is defined as follows (the reorientation energy

for electron conduction can be calculated similarly by considering the anionic state instead of the cationic state).

$$\lambda = (E_{+}^{*} - E_{+}) + (E_{0}^{*} - E_{0}) \tag{4}$$

Here, $E_0$ and $E_+$ represent the energies of the neutral and cationic species at their respective optimized geometric structures. $E_0^*$ denotes the energy of the neutral state at the geometric structure of the cationic species, while $E_+^*$ represents the energy of the cationic state at the geometric structure of the neutral species. In other words, $E_+^* - E_+$ corresponds to the energy released during the structural relaxation process when a neutral molecule accepts a hole and transitions to the optimal geometric structure of the cationic state. Similarly, $E_0^* - E_0$ represents the energy released when a cationic molecule releases a hole and transitions to the optimal geometric structure of the neutral state. Furthermore, $\lambda$ is a constant that does not depend on the carrier hopping pathway.

By substituting the calculated value of $\lambda$ into Equation (1), it becomes possible to simulate the absolute value of carrier mobility. However, in the case of **Ni(4OPr)**, the central metal Ni requires a different basis set compared to other atoms, which significantly increases the computational cost for calculating $\lambda$ compared to organic molecules that do not contain metal atoms. As a result, we determined that the reliability of this calculation could not be fully ensured. Furthermore, this study focuses on the experimental observation of anisotropic carrier transport properties, and the simulation data is used solely as supplementary material for interpreting experimental results. Therefore, instead of calculating the reorganization energy, we simulated only the relative carrier mobility for different orientations based on structural information and intermolecular interactions. In the simulation data presented in this paper regarding the in-plane angular dependence of mobility, we provide graphs where the maximum mobility is normalized to 1.

## 2. Calculation of the Crystal Orbitals of Ni(4OPr)

We calculated the crystal orbitals (Figures 4 and S2) using the first-principles software OpenMX (Version 3.9),[33–37] with optimized local basis functions and pseudopotentials. In the calculations, the crystallographic coordinate by the single-crystal X-ray structural analysis was used as the single point without further optimization. The following basis sets were used: H: H6.0-s2p1, C: C6.0-s2p2d1, O: O6.0-s2p2d1, S: S7.0-s2p2d1f1, Ni: Ni6.0H-s3p2d1 (The first part indicates the atom, the number after it is the cutoff radius (in Bohr) used in the confinement scheme, and the notation after the hyphen indicates how many optimized radial functions are adopted for each orbital type, e.g., s2p2d1 for two s, two p, and one d radial function.). The radial function was optimized using the variational optimization method. For the valence electrons in the pseudopotential (PP), 1s was used for hydrogen; 2s and 2p for carbon and oxygen; 3s and 3p for sulfur; and 3s, 3p, 3d, and 4s for Ni. All PPs and pseudo-atomic orbitals (PAOs) used in this study were obtained from the OpenMX website database (2019)[33] and benchmarked using the delta gauge method. For numerical integration and the solution of the Poisson equation using the FFT method, a real-space grid approach was employed, with an energy cutoff of 220 Ryd. The Brillouin zone integration was performed on a $1 \times 2 \times 2$ $k$-grid, and the Fermi-Dirac distribution function at 300 K was adopted as the smeared occupancy function. The exchange-correlation functional was treated using the generalized gradient approximation (GGA) proposed by Perdew, Burke, and Ernzerhof.[38] The simulation indicates that crystal orbitals, especially the LUMO-derived crystal orbital (lowest unoccupied crystal orbital, LUCO) at the G point (Figures 4c,f, and S2), exhibit two types of significant transverse intermolecular in-phase overlap between neighboring columns, thereby enhancing substantial transverse transfer integrals. Such significant interactions are absent in the HOMO-derived crystal orbital (the highest occupied crystal orbital, HOCO) at the G point (Figures 4b, e).

Notably, these principal conduction paths derived from the crystal orbitals (red dashed bold and thin arrows in S2b,c) are shorter than the electron hopping paths (black solid arrows in Figure S2a) assumed for single-hopping events in the adjacent column direction (refer to Figure 1 for details). This difference implies that the mobility calculation, by assuming longer electron hopping distances, effectively overestimates the electron transfer distance. Consequently, this may contribute to an overestimation of electron mobility in the transverse direction in the calculations, which could account for the observed discrepancy in the degree of electron mobility anisotropy.

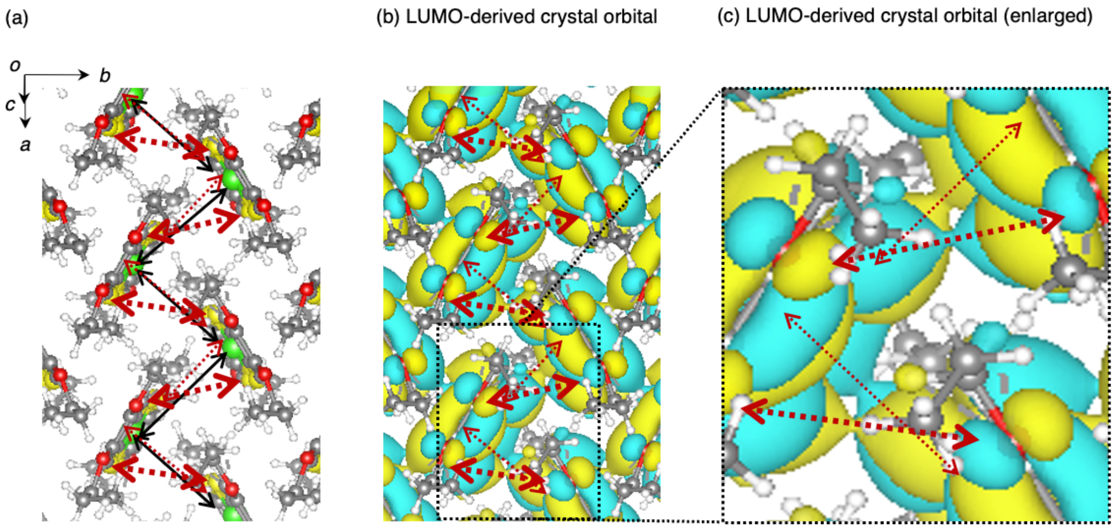


**Figure S2**. Crystal orbitals and electron transport within adjacent molecular stacking columns of **Ni(4OPr)**. (a) The black solid arrows represent the electron hopping paths assumed in the simulation, connecting the centers of gravity of molecules (refer to Figure S1 for details). The red dashed bold and thin arrows indicate the two types of principal conduction paths derived from the crystal orbitals (see Figures 4c,f and S2b,c). Note that the electron hopping distance per event differs between the paths indicated by black and red arrows. (b, c) LUMO-derived crystal orbital (the lowest unoccupied crystal orbital, LUCO) at the Γ point. The red dashed bold and thin arrows highlight two types of transverse intermolecular in-phase overlap between neighboring columns, which are considered to contribute strongly to transverse electron transport. (c) Enlarged view of (b).

## 3. FET Fabrication and Characterization

We synthesized the neutral **Ni(4OPr)** complex following a previously reported method.[21] As substrates, we used heavily *n*-doped Si/$SiO_2$ wafers with a 300 nm thermal oxide layer, cut into 1 cm square chips. Each chip was cleaned by ultrasonic treatment in acetone, deionized water, and 2-propanol (10 min each), followed by 20 min of UV-ozone treatment (using a TECHNOVISION Model208). We then deposited parylene-C via chemical vapor deposition (Specialty Coating Systems PDS 2010 LABCOTER) using dichlorodi-*p*-xylylene as a precursor.

**Ni(4OPr)** crystalline thin films were formed on these parylene-treated substrates via blade coating19 (Opto Sigma Stepping Motor Drive SHOT-302GS). The blade-coating conditions were optimized from our original report[21]: we used a higher solution concentration of 1.6 mg $mL^{-1}$ in *o*-dichlorobenzene and raised the blade speed to 6 cm $h^{-1}$. The substrate temperature was

maintained at 80 °C, as before. Although multiple grain boundaries appeared on the substrate, we isolated a single crystalline domain (Typical domain size: approximately 500 × 500 $\mu m^2$; see Figure S5 for the image) per substrate for subsequent GIWAXS analysis and FET measurements, removing all other domains. The surface roughness of the thin film was characterized by atomic force microscopy (AFM; MFP-3D in tapping mode; Asylum Research; Figure S3). Angle-dependent WAXS exhibited sharp diffraction peaks (Figures 3a,b and S6) confined to a single azimuthal orientation, indicating the formation of single-crystalline domains. Consistently, AFM over scan areas of 625 $\mu m^2$ (25 µm × 25 µm; Figure S3b) and 900 $\mu m^2$ (30 µm × 30 µm; Figure S3e) revealed a step-terrace morphology with individual step heights quantized at the projection of the *a*-axis length (~1.9 nm) onto the surface normal. As shown in Figure S3c,f, the line profiles are plotted with z-scales of ±6 nm (25 µm) and ±3 nm (30 µm), corresponding to overall height excursions of ~12 nm and ~6 nm across the scan—i.e., only a few monolayer steps. No pronounced non-periodic height fluctuations are observed, which is characteristic of wide-terrace, layer-by-layer growth.

We then thermally evaporated approximately 40 nm-thick gold electrodes (source/drain) through a metal shadow mask. The electrode pattern consisted of 12 circularly arranged segments at 30° intervals, allowing in-plane angular measurements in 30° increments. The channel length was approximately 50 µm, verified by optical microscopy to correct for minor errors due to mask lift or warping. The transistor characteristics (transfer and output curves) were measured with a Keithley 4200 semiconductor characterization system.

We calculated the carrier mobility ($\mu$) in the saturation region from the equation: $I_D = (\mu W C_i/2L)/(V_G - V_{th})^2$, where $I_D$ is the drain current, $W$ is the channel width, $L$ is the channel length, $C_i$ is the capacitance per unit area of the dielectric layer, $V_G$ is the gate voltage, and $V_{th}$ is the threshold voltage. Vacuum measurements were performed at approximately $10^{-3}$ Pa using a Pfeiffer Vacuum HiCube 80 Eco turbo pump. We defined Channel 12 to be parallel to the solution-sweep (film-growth) direction (corresponding to $\varphi' = 90$) and numbered channels 1 to 11 clockwise from there. FET measurements proceeded in the order of channel alignment, rotating clockwise.

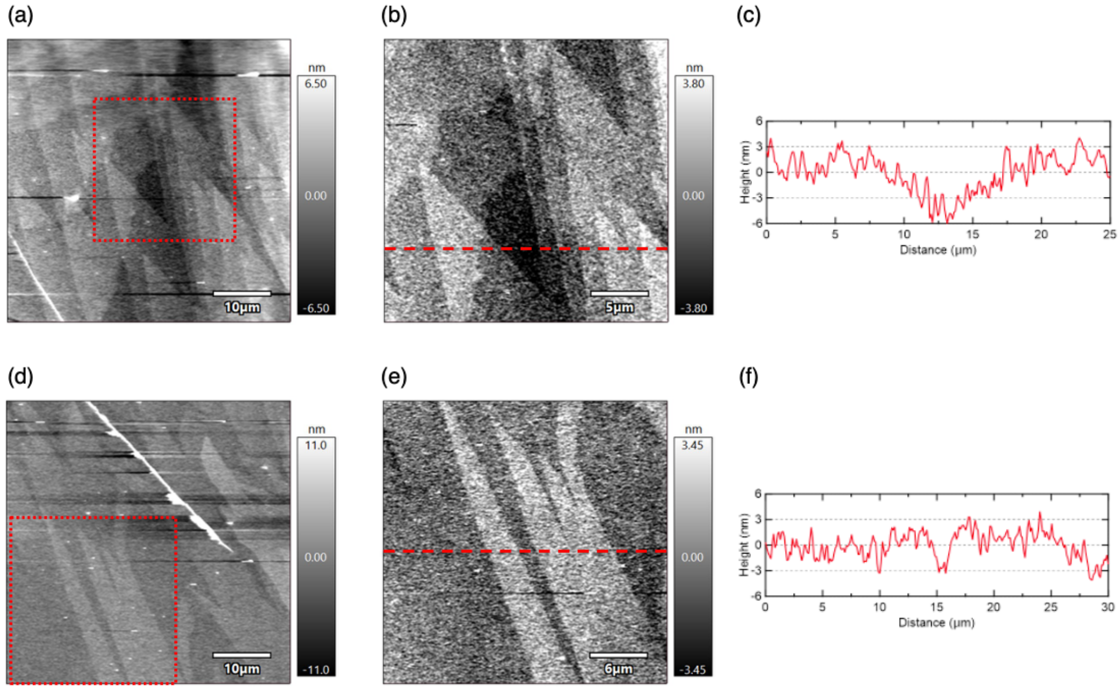


**Figure S3**. Atomic force microscopy (AFM) images of a blade-coated Ni(4OPr) thin film. (a) 50 μm × 50 μm scan area. (b) Enlarged view of the dotted square in (a) showing a 25 μm × 25 μm scan area. (c) Height profile along the red dashed line in (b). (d) 50 μm × 50 μm scan area. (e) Enlarged view of the dotted square in (d) showing a 30 μm × 30 μm scan area. (f) Height profile along the red dashed line in (e).

## 4. Determination of the In-Plane Molecular Orientation in FET Thin Films

The in-plane molecular orientation of the crystalline thin film, which serves as the active layer of the FET subject to characteristic evaluation, was investigated using a GIWAXS apparatus. The crystalline thin film used in this experiment consisted of a single crystalline domain with an area of approximately 500 μm square. While this area was sufficient for FET fabrication, determining the in-plane crystallographic orientation required the use of high-intensity X-rays. Therefore, in this experiment, we utilized the GIWAXS apparatus at the Photon Factory (PF) in the High Energy Accelerator Research Organization (KEK) at Tsukuba, Ibaraki, Japan. The beamline used was BL10C, and the detector was a PILATUS3 2M manufactured by DECTRIS. The sample-to-detector distance was approximately 246 mm, which was calibrated by the standard sample (silver behenate). An automatic rotation stage was incorporated into the apparatus, allowing X-ray irradiation while rotating the sample (see Figure S4). Diffraction spots were extracted from the GIWAXS profiles obtained at each X-ray irradiation angle. By comparing these with a simulated powder pattern based on single-crystal X-ray structure analysis results, the Miller indices (*hkl*) of

each diffraction spot were assigned (Table S1). Since the X-ray wavelengths used in the GIWAXS measurements and single-crystal X-ray diffraction measurements differed (1.5 Å and 0.71073 Å, respectively), Q-values were used for indexing.

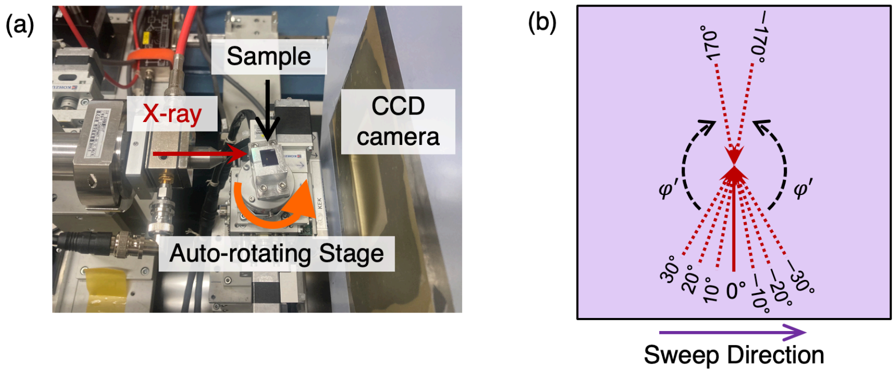


**Figure S4**. GIWAXS instrument with auto-rotating stage (a) and definition of X-ray incidence angle $\varphi'$ (b).

**Table S1**. Miller index (*hkl*) and *d*-spacing, X-ray intensity ($|F|^2$, where F is the scattering amplitude), and Q-value for each peak extracted from the **Ni(4OPr)** powder pattern. In this table, the peaks are listed from the top in order of decreasing Q-value. Powder patterns are simulated based on the results of X-ray single-crystal structural analysis.

| *h* | *k* | *l* | *d*-spacing | $\|F\|^2$ | Q |
|---|---|---|---|---|---|
| 1 | 0 | 0 | 18.1491 | 16701.2 | 0.34619818 |
| 2 | 0 | 0 | 9.07453 | 138.62 | 0.69239788 |
| 1 | 1 | 0 | 7.47525 | 2253.92 | 0.84053180 |
| 2 | 1 | 0 | 6.08541 | 135.75 | 1.03249991 |
| 3 | 0 | 0 | 6.04969 | 934.853 | 1.03859624 |
| 0 | 1 | 1 | 5.96769 | 4566.23 | 1.05286724 |
| 1 | 1 | −1 | 5.94533 | 344.955 | 1.05682701 |
| 1 | 1 | 1 | 5.42808 | 19940.4 | 1.15753366 |
| 2 | 1 | −1 | 5.37802 | 6408.65 | 1.16830828 |
| 3 | 1 | 0 | 4.86890 | 2948.15 | 1.29047327 |
| 2 | 1 | 1 | 4.66904 | 19819.6 | 1.34571246 |
| 3 | 1 | −1 | 4.61611 | 799.346 | 1.36114289 |
| 4 | 0 | 0 | 4.53727 | 5641.74 | 1.38479423 |
| 1 | 0 | −2 | 4.46030 | 70.7971 | 1.40869119 |

| | | | | | |
|---|---|---|---|---|---|
| 0 | 0 | 2 | 4.34871 | 366.679 | 1.44483888 |
| 2 | 0 | –2 | 4.31432 | 6863.91 | 1.45635588 |
| 0 | 2 | 0 | 4.10170 | 15361.6 | 1.53184906 |
| 1 | 0 | 2 | 4.03032 | 11690.7 | 1.55897926 |
| 3 | 0 | –2 | 3.97603 | 38.1515 | 1.58026607 |
| 4 | 1 | 0 | 3.97042 | 383.102 | 1.58249891 |
| 3 | 1 | 1 | 3.95669 | 4884.49 | 1.58799029 |
| 4 | 1 | –1 | 3.91160 | 0.0013326 | 1.60629546 |
| 5 | 0 | 0 | 3.62981 | 1372.11 | 1.73099565 |
| 2 | 0 | 2 | 3.61978 | 4218.82 | 1.73579204 |
| 4 | 0 | –2 | 3.56102 | 543.853 | 1.76443415 |
| 4 | 1 | 1 | 3.37193 | 8.80052 | 1.86337952 |
| 5 | 1 | –1 | 3.33600 | 220.289 | 1.88344883 |
| 5 | 1 | 0 | 3.31938 | 0.250214 | 1.89287918 |
| 3 | 0 | 2 | 3.20836 | 622.087 | 1.95837914 |
| 5 | 0 | –2 | 3.15388 | 474.406 | 1.99220811 |
| 6 | 0 | 0 | 3.02484 | 1299.16 | 2.07719592 |
| 5 | 1 | 1 | 2.90989 | 399.086 | 2.15925183 |
| 6 | 1 | –1 | 2.88163 | 1754.22 | 2.18042750 |
| 4 | 0 | 2 | 2.83926 | 1739.03 | 2.21296581 |
| 6 | 1 | 0 | 2.83806 | 1969.1 | 2.21390151 |
| 6 | 0 | –2 | 2.79203 | 1503.88 | 2.25040036 |

**5. Correlation between FET characteristics under vacuum and crystallographic orientation in the channel directions**

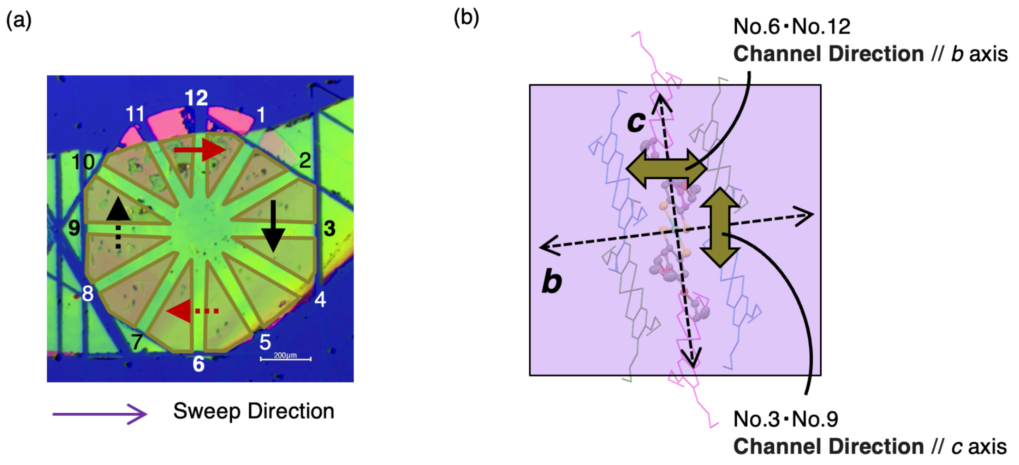

**Figure S5**. (a) The relationship between the channel directions used for measurement under vacuum. (b) The crystallographic directions in the FET.

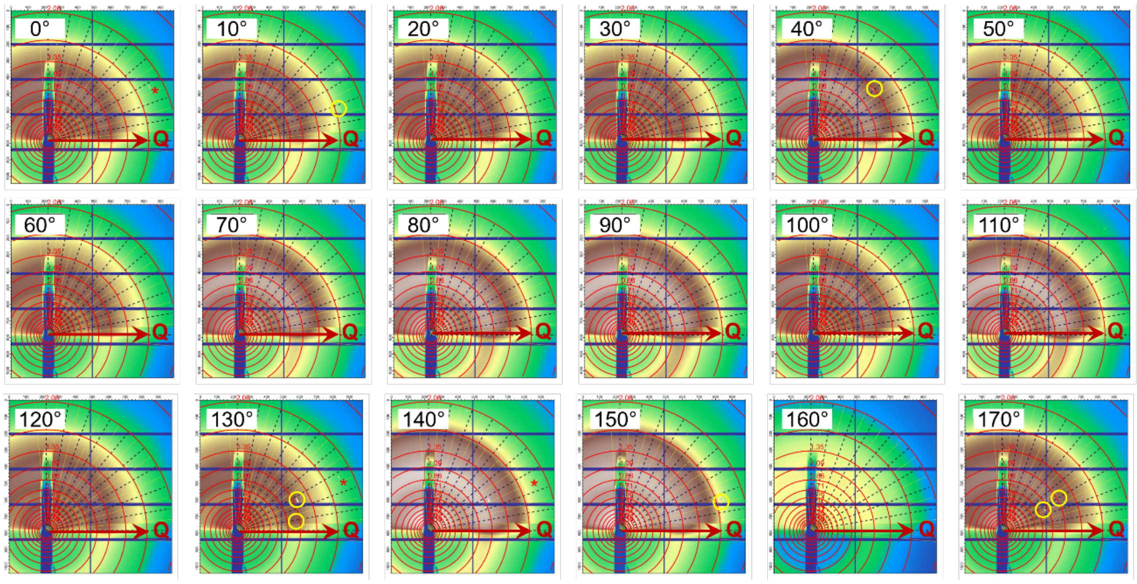


**Figure S6**. GIWAXS profiles at each X-ray incidence angles $\varphi'$. Yellow circles highlight diffraction spots used for in-plane orientation determination. Asterisk indicates diffraction spot derived from the $Si/SiO_2$ substrate.

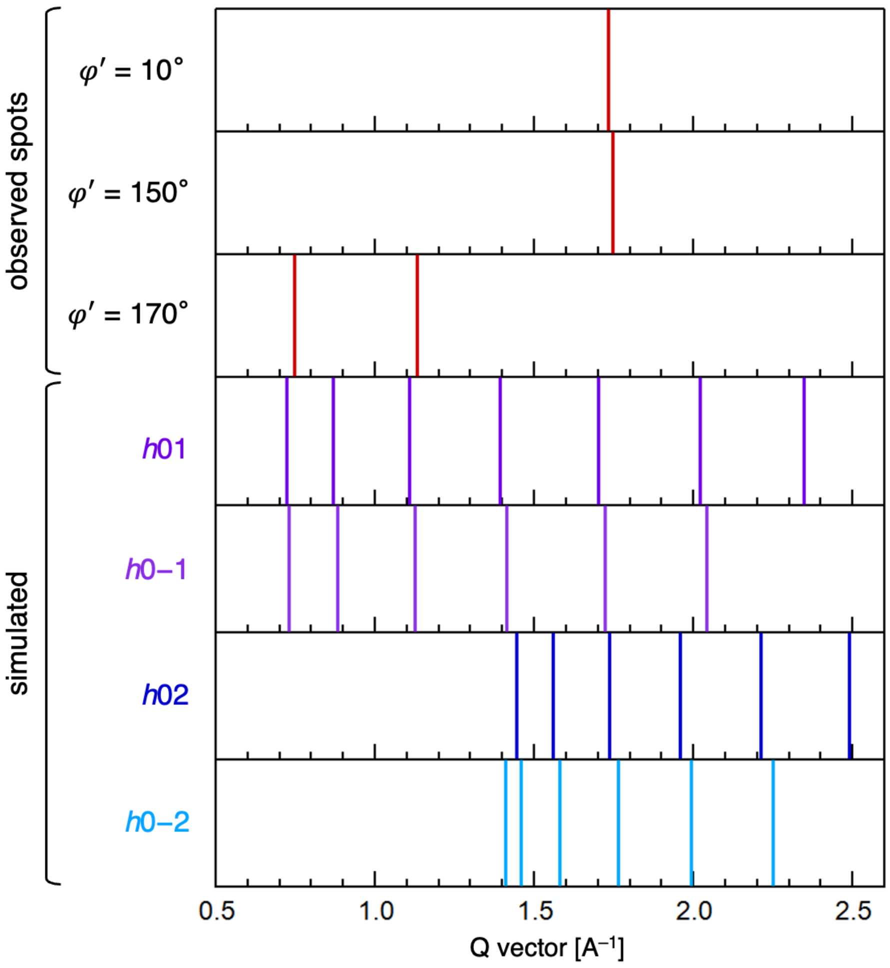


**Figure S7**. Attribution of the *h*0±1 and *h*0±2 diffraction spots found at incidence angles of 10°, 150° and 170° based on their Q values.

## 6. Correlation between FET characteristics in air and crystallographic orientation in the channel directions

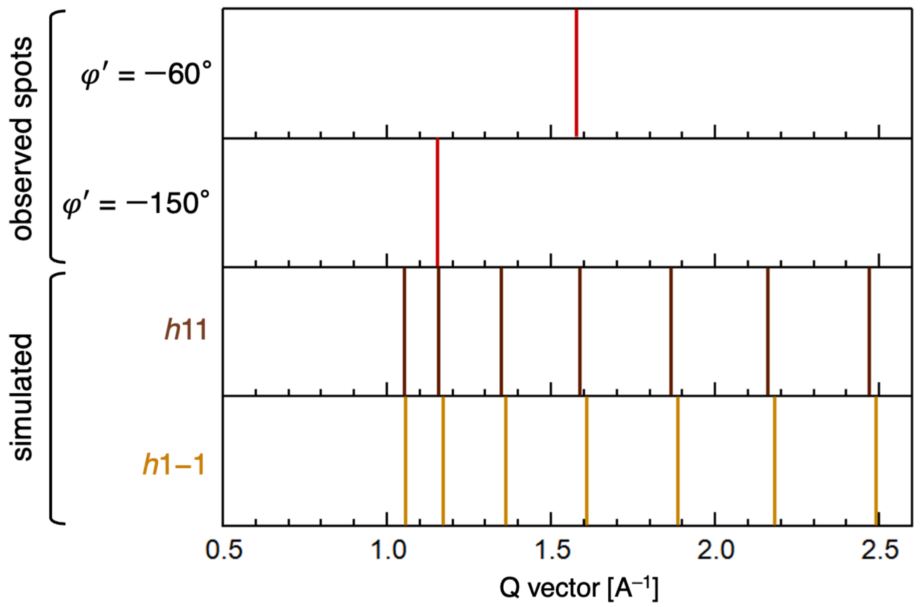


**Figure S8**. Attribution of the *h*0±1 and *h*0±2 diffraction spots found at incidence angles of –60°, and –150° based on their Q values.

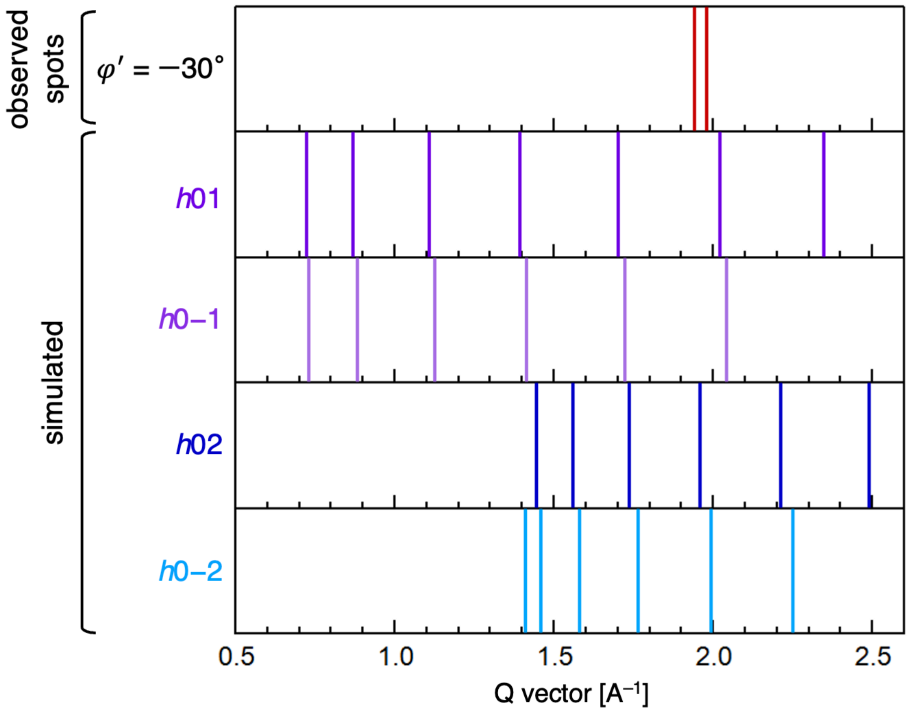


**Figure S9**. Attribution of the *h*0±1 and *h*0±2 diffraction spots found at incidence angle of –30° based on their Q values.

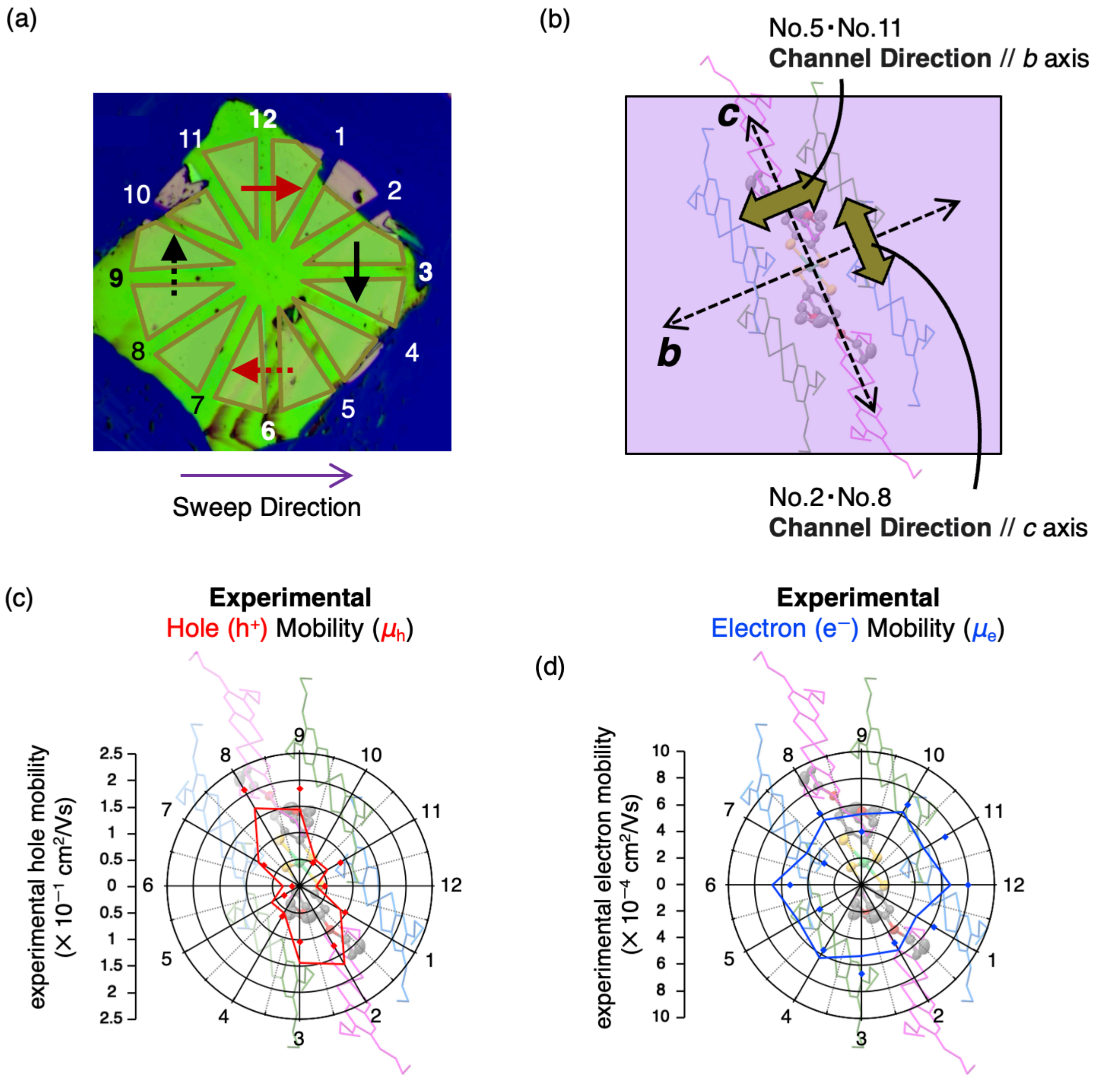


**Figure S10**. (a) Attribution of the diffraction spots in the GIWAXS profile based on the powder pattern simulated from the X-ray single-crystal structural analysis. (b) Relationship between the channel directions and crystallographic directions in the FET used for measurement. (c) Experimental in-plane angular dependence of hole mobility measured in air. The highest hole mobility was $2.1 \times 10^{-1}$ $cm^2$ $V^{-1}$ $s^{-1}$ with on/off ratio of approximately $10^5$ was observed at Channel 8. The ratio of maximum to minimum hole mobility was 14. (d) Experimental in-plane angular dependence of electron mobility measured in air. The highest electron mobility was $8.1 \times 10^{-4}$ $cm^2$ $V^{-1}$ $s^{-1}$ with on/off ratio of approximately $10^4$ was observed at Channel 12. The ratio of maximum to minimum hole mobility was 2.5. In the graphs (c) and (d), each data point shows the mobility observed in that channel, and the lines connect the averaged mobility of two channels oriented in the same direction (e.g., 12–6, 1–7, 2–8, etc.).

**Table S2**. Carrier mobilities, threshold voltages ($V_{th}$) and on/off ratios of each channel in the above FET in air.

| Channel No. | Hole transport | | | Electron transport | | |
|---|---|---|---|---|---|---|
| | Mobility ($cm^2 V^{-1} s^{-1}$) | $V_{th}$ (V) | On/off ratio | Mobility ($cm^2 V^{-1} s^{-1}$) | $V_{th}$ (V) | On/off ratio |
| 1 | $9.9 \times 10^{-2}$ | 47 | $10^4$ | $6.3 \times 10^{-4}$ | 110 | $10^3$ |
| 2 | $1.3 \times 10^{-1}$ | 41 | $10^5$ | $5.0 \times 10^{-4}$ | 114 | $10^3$ |
| 3 | $1.1 \times 10^{-1}$ | 41 | $10^4$ | $6.7 \times 10^{-4}$ | 116 | $10^3$ |
| 4 | $6.6 \times 10^{-2}$ | 35 | $10^5$ | $5.7 \times 10^{-4}$ | 103 | $10^2$ |
| 5 | $3.4 \times 10^{-2}$ | 34 | $10^5$ | $3.7 \times 10^{-4}$ | 105 | $10^3$ |
| 6 | $1.5 \times 10^{-2}$ | −1.9 | $10^6$ | $5.4 \times 10^{-4}$ | 69 | $10^3$ |
| 7 | $7.9 \times 10^{-2}$ | 45 | $10^4$ | $3.2 \times 10^{-4}$ | 120 | $10^3$ |
| 8 | $2.1 \times 10^{-1}$ | 42 | $10^5$ | $6.3 \times 10^{-4}$ | 125 | $10^3$ |
| 9 | $1.9 \times 10^{-1}$ | 40 | $10^5$ | $4.0 \times 10^{-4}$ | 118 | $10^4$ |
| 10 | $5.2 \times 10^{-2}$ | 40 | $10^5$ | $7.0 \times 10^{-4}$ | 121 | $10^3$ |
| 11 | $8.9 \times 10^{-2}$ | 24 | $10^6$ | $7.3 \times 10^{-4}$ | 113 | $10^3$ |
| 12 | $4.8 \times 10^{-2}$ | 56 | $10^3$ | $8.1 \times 10^{-4}$ | 123 | $10^4$ |

Highly anisotropic hole mobility and relatively isotropic electron mobility were observed in air, consistent with the simulation (Figure 2b,e). The highest hole mobility in air ($2.1 \times 10^{-1}$ $cm^2 V^{-1} s^{-1}$, Table S2) far exceeds our previously reported maximum of $2.0 \times 10^{-3}$ $cm^2 V^{-1} s^{-1}$.[18] This significant improvement is primarily attributed to the optimization of the channel orientation, rather than solely to improved thin film growth conditions. Indeed, the ratio of maximum to minimum hole mobility is approximately 14 (Table S2) under ambient conditions, consistent with the notion that holes conduct predominantly in the intracolumn direction, while the earlier work had channels aligned parallel to the film-growth direction, which was suboptimal for hole conduction.

Meanwhile, electron transport under ambient conditions shows overall lower mobility and slightly higher threshold voltages compared to vacuum, indicating stronger environmental effects on n-type conduction. However, the ratio between maximum and minimum electron mobilities remains approximately 2.5 (Table S2), reproducing the vacuum trend of more isotropic electron transport than holes.
